# Neutralization Titers in Vaccinated Patients with SARS-CoV-2 Delta Breakthrough Infections

**DOI:** 10.1128/mbio.01996-22

**Published:** 2022-08-04

**Authors:** Jing Zou, Xuping Xie, Mingru Liu, Pei-Yong Shi, Ping Ren

**Affiliations:** a Department of Biochemistry and Molecular Biology, University of Texas Medical Branch, Galveston Texas, USA; b Department of Pathology, University of Texas Medical Branch, Galveston Texas, USA; c Institute for Human Infection and Immunity, University of Texas Medical Branch, Galveston, Texas, USA; d Sealy Institute for Drug Discovery, University of Texas Medical Branch, Galveston, Texas, USA; e Institute for Translational Sciences, University of Texas Medical Branch, Galveston, Texas, USA; f Sealy Institute for Vaccine Sciences, University of Texas Medical Branch, Galveston, Texas, USA; g Sealy Center for Structural Biology & Molecular Biophysics, University of Texas Medical Branch, Galveston, Texas, USA; University of Pennsylvania

**Keywords:** COVID-19, SARS-CoV-2, breakthrough infection, antibody neutralization, vaccine, variants of concern, vaccine booster

## Abstract

The continuous emergence of SARS-CoV-2 variants with increased transmission and immune evasion has caused breakthrough infections in the vaccinated population. It is important to determine the threshold of neutralizing antibody titers (NT_50_) that permit breakthrough infections in humans. Here, we tested the neutralization titers of vaccinated patients who contracted Delta variant. All 64 patients with Delta breakthrough infections exhibited NT_50_ of less than 70. When the breakthrough sera were tested against USA-WA1/2020 (a strain isolated in late January 2020), 82.8%, 15.6%, and 1.6% of them had the NT_50_ ranges of <20, 20 to 50, and 50 to 69, respectively. When the same breakthrough sera were tested against Delta-spike SARS-CoV-2, 68.7%, 26.6%, and 4.7% of them had the NT_50_ ranges of <20, 20 to 50, and 50 to 69, respectively. Overall, the results suggest NT_50_ of 70 as a potential neutralizing threshold required to prevent Delta breakthrough infections. These clinical laboratory results have implications in vaccine strategy and public health policy.

## OBSERVATION

The COVID-19 pandemic is umpired by two dynamic factors: (i) the continuous emergence of SARS-CoV-2 variants with improved transmission and/or immune evasion and (ii) the waning immunity post vaccination and infections (1). This is exemplified by the two recent surges of Delta and Omicron variants, which caused many breakthrough infections in vaccinated individuals. Because antibody neutralization is a key contributor to vaccine protection against symptomatic infection and severe disease (2), it is important to define the neutralization levels in vaccinated individuals who contracted breakthrough infections. Such information is essential to guide vaccine strategy and policy. Here, we characterized the antibody neutralization in vaccinated patients when they acquired Delta variant infections.

**FIG 1:**
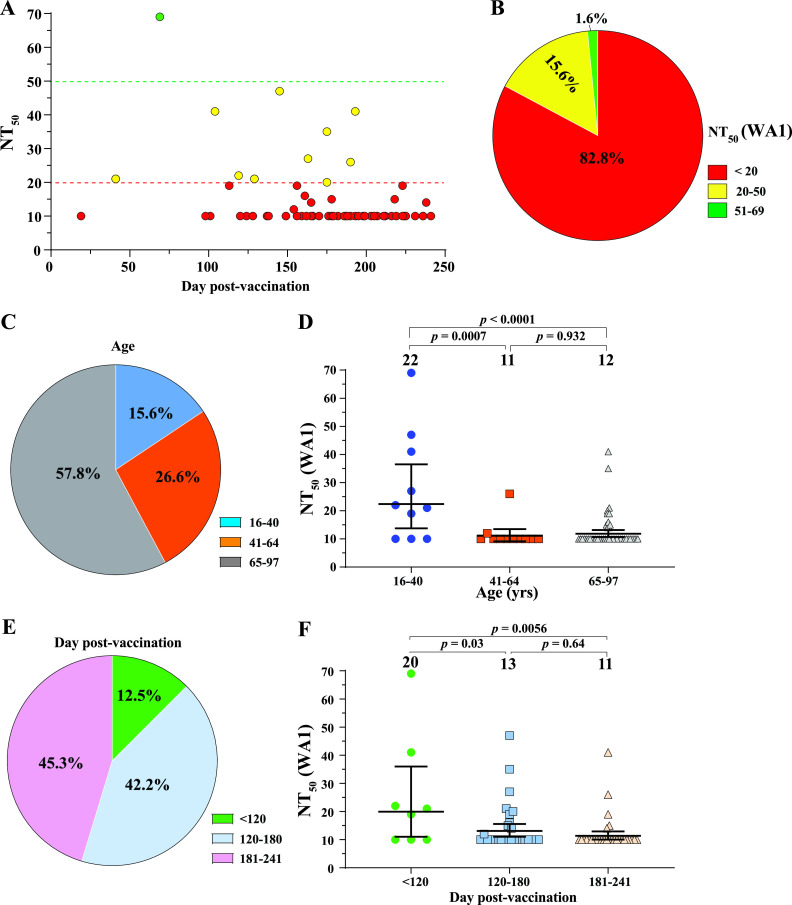
NT_50_ analysis against USA-WA1/202 SARS-CoV-2. A panel of 64 sera, collected from Delta breakthrough patients, were measured for antibody neutralization titers (NT_50_) against mNeonGreen USA-WA1/202. All patients were immunized with two doses of Pfizer or Moderna vaccine or one dose of J&J vaccine. All sera were taken 0 to 5 days before viral RNA-positive test results. The sera were heat-inactivated at 56°C for 30 min before the neutralization testing. All patient information and NT_50_ values are detailed in Table S1. (A) Plot of NT_50_ values versus days after dose 2 of Pfizer or Moderna vaccine or after 1 dose of J&J vaccine. Each data point represents the geometric mean of NT_50_ for one serum tested in duplicate assays. Different colors represent different NT_50_ ranges. Samples with no detectable neutralizing activities were plotted as 10 for calculation purpose. (B) Pie presentation of different NT_50_ ranges. (C) Age distributions. (D) Plot of NT_50_ of different age groups. (E) Distribution of breakthrough percentages versus days post vaccination. (F) Plot of NT_50_ versus days post vaccination. In D and F, the bar heights and the numbers above indicate geometric mean titers. The whiskers indicate 95% confidence intervals. Statistical analysis was performed using the one-way ANOVA with Tukey’s correction for multiple-comparison test.

To determine the neutralization titers (NT_50_) in breakthrough patients when they were infected with Delta variant, we collected sera from 64 patients who were vaccinated and subsequently contracted breakthrough infections. The use of human sera for the described research was reviewed and approved by the University of Texas Medical Branch (UTMB) Institutional Review Board (IRB number 20–0070). The deidentified human sera were collected from vaccinated patients who presented COVID-19 symptoms. Table S1 summarizes the patient information and their NT_50_ values. All patients were immunized with two doses of Pfizer or Moderna vaccine or one dose of J&J vaccine. Breakthrough infections were confirmed by positive viral RNA tests. Although the genotypes of individual infecting viruses were not determined by sequencing, they were most likely Delta variant because all infections had occurred from late July to October 2021, when Delta was 100% prevalent in our patient population based on the local SARS-CoV-2 surveillance system (at the University of Texas Medical Branch) and about 98% prevalent in the United States (https://covid.cdc.gov/covid-data-tracker/#variant-proportions). All sera were taken 0 to 5 days before the positive viral nucleic acid tests and within 4 days of symptom onset. We determined the NT_50_ of each serum using a well-established mNeonGreen reporter USA-WA1/2020 SARS-CoV-2 (3). This neutralization assay has been reliably used to support the BNT162b2 vaccine development (4–6). The NT_50_ value was defined as the interpolated reciprocal of the dilution yielding a 50% reduction in mNeonGreen-positive cells. Each specimen was tested in duplicates and the geometric mean of the duplicate results is presented. The first serum dilution of the neutralization test was 1:20. The NT_50_ values of specimens with no detectable neutralizing activities at 1:20 dilution were treated as 10 for plot and calculation purposes (Table S1). The overall results reveal three observations. First, all breakthrough patients had low NT_50_s of <70 (Fig. 1A). About 82.8%, 15.6%, and 1.6% of the breakthrough patients exhibited the NT_50_ ranges of <20, 20 to 50, and 51 to 69, respectively (Fig. 1B). The results suggest NT_50_ of 70 as a potential neutralizing threshold required to prevent Delta breakthrough infections. In support of our human results, a previous study reported NT_50_ of 50 as the minimal neutralization level required to protect nonhuman primates from SARS-CoV-2 infection (7). Second, senior people appeared to be more vulnerable to breakthrough infections. Approximately 15.6%, 26.6%, and 57.8% of the breakthrough cases were in the age groups of 16 to 40, 41 to 64, and 65 to 97, respectively (Fig. 1C). However, the NT_50_ differences among the three age groups are not all statistically significant (Fig. 1D). Third, 87.5% of the breakthrough patients had received two doses of Pfizer or Moderna vaccine or one dose of J&J vaccine for more than 120 days (Fig. 1E). However, this observation was not always statistically correlated with the NT_50_ differences among different time frames post vaccination (Fig. 1F).

10.1128/mbio.01996-22.1 TABLE S1 Serum information and NT_50_ values. Serum information, including age, gender, ethnicity of patient with vaccine type, and serum collection time (days post positive NAAT and days post last vaccination), are indicated. Neutralization titers against WA1 and Delta are shown. Download Table S1, DOCX file, 0.02 MB. Copyright © 2022 Zou et al. 2022 Zou et al. https://creativecommons.org/licenses/by/4.0/ This content is distributed under the terms of the Creative Commons Attribution 4.0 International license.

**FIG 2:**
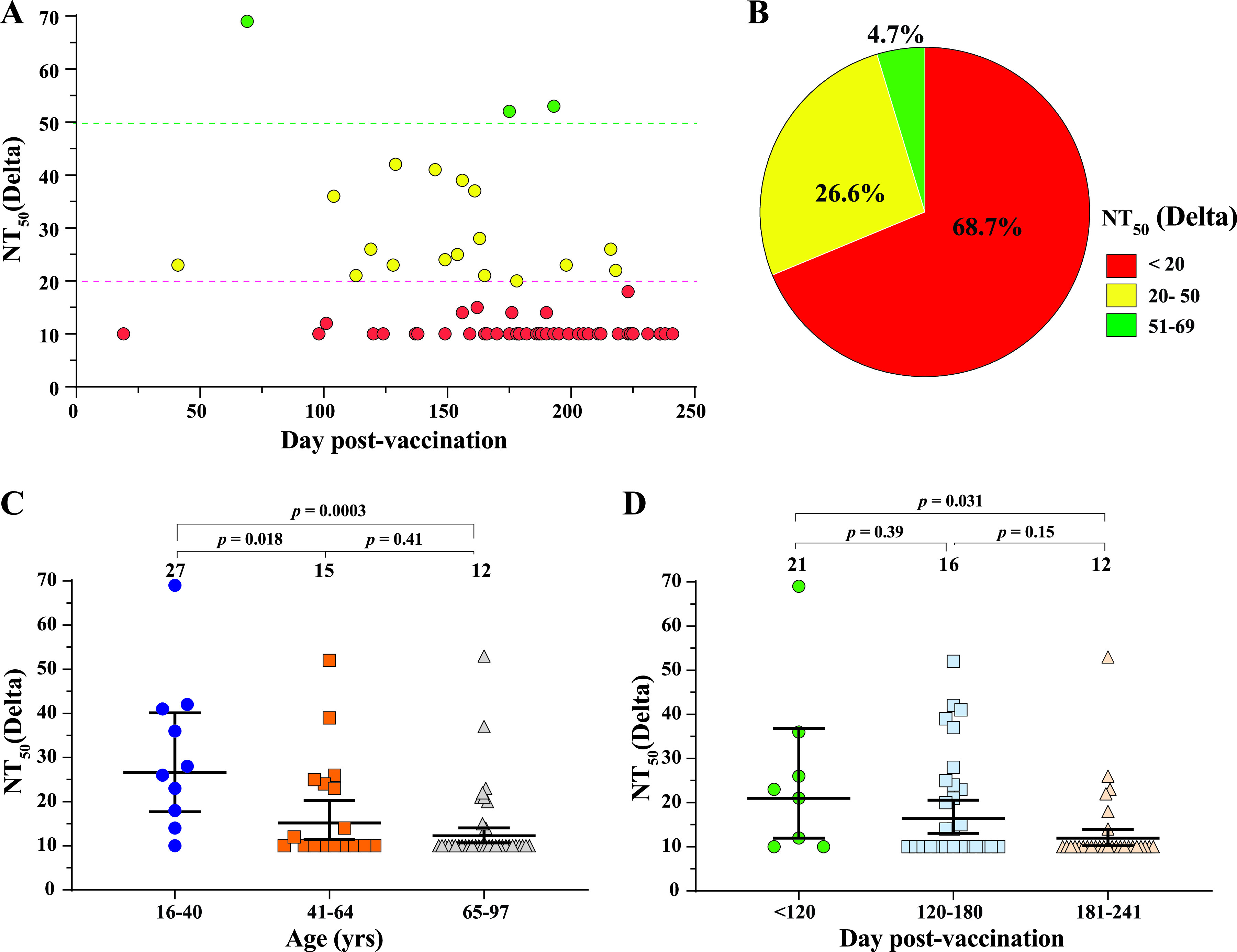
NT_50_ analysis against Delta-spike SARS-CoV-2. The same sera from Fig. 1 were measured for NT_50_ values against mNeonGreen Delta-spike SARS-CoV-2. Delta-spike SARS-CoV-2 contains the complete spike gene from the Delta variant in the backbone of mNeonGreen USA-WA1/2020 SARS-CoV-2. The NT_50_ values are summarized in Table S1. The NT_50_ values were measured and presented as described in Fig. 1. (A) Plot of NT_50_ values against Delta-spike SARS-CoV-2. (B) Pie presentation of different NT_50_ ranges. (C) Plot of NT_50_ against Delta-spike SARS-CoV-2 of different age groups. (D) Plot of NT_50_ against Delta-spike SARS-CoV-2 versus days post vaccination. In C and D, the numbers above indicate geometric mean titers. The whiskers indicate 95% confidence intervals. Statistical analysis was performed with the one-way ANOVA with Tukey’s correction for multiple-comparison test.

The above analysis was performed on the NT_50_ values against the original mNeonGreen USA-WA1/2020 (3). To directly test the NT_50_ values against Delta spike, we engineered the complete spike gene from the Delta variant into the backbone of mNeonGreen USA-WA1/2020, resulting in Delta-spike SARS-CoV-2. The construction of the Delta-spike SARS-CoV-2 was previously reported (8, 9). We tested the entire serum panel for NT_50_ values against the Delta-spike SARS-CoV-2 (Table S1). Compared with the original USA-WA1/2020 (Fig. 1), the Delta-spike SARS-CoV-2 results revealed similar observations (Fig. 2). All breakthrough sera had NT_50_s of <70 against Delta-spike SARS-CoV-2 (Fig. 2A). Approximately 68.7%, 26.6%, and 4.7% of the breakthrough sera exhibited the NT_50_ ranges of <20, 20 to 50, and 51 to 69, respectively (Fig. 2B). The NT_50_ differences among the three age groups (Fig. 2C) or the NT_50_ differences among the three time frames post vaccination (Fig. 1D) are not always statistically significant.

We previously reported that at 8 months post dose-2 of BNT162b2 vaccine, the neutralization titers against USA-WA1/2020 were 83 and 41 for age groups of 18 to 55 and 65 to 85 years old, respectively (4), suggesting that both age groups, particularly the senior age group, are susceptible to Delta breakthrough infections. The previous result, together with the current data, support the vaccine booster strategy after 6 months of two doses of BNT162b2.

The current study has three weaknesses. The first weakness is even though the serum specimens were collected on 0 to 5 days before viral RNA-positive tests, these sera may already contain anamnestic antibodies produced during the presymptomatic period of breakthrough infections, which may lead to an overestimated NT_50_ threshold for breakthrough infections. The second weakness is the small sample size of the study; more breakthrough patient specimens are needed to bolster our conclusion. The third weakness is the NT50 value of vaccinated individuals who resisted Delta breakthrough infections are unknown. Despite the weaknesses, the current results gave a glimpse of the neutralization status when the vaccinated patients contracted Delta breakthrough infections.

Compared with Delta, the newly emerged Omicron is significantly less susceptible to neutralization by vaccinated or non-Omicron infected human sera (10–13). The reduced neutralization susceptibility, combined with the increased transmissibility of Omicron, may have accounted for the Delta-to-Omicron variant replacement and high breakthrough infections observed in the current Omicron surge. Similar clinical studies are needed to understand the threshold of neutralization required to protect humans from Omicron breakthrough infections. Laboratory investigations, together with the real-world vaccine effectiveness, have enabled FDA to recommend a bivalent vaccine strategy that includes both the original spike and the current prevalent Omicron BA.4/5 spike.
