# Correction for Zhou et al., “Neutralization Titers in Vaccinated Patients with SARS-CoV-2 Delta Breakthrough Infections”

**DOI:** 10.1128/mbio.03108-22

**Published:** 2022-12-05

**Authors:** Jing Zou, Xuping Xie, Mingru Liu, Pei-Yong Shi, Ping Ren

## AUTHOR CORRECTION

Volume 13, no. 4, e01996-22, 2022, https://doi.org/10.1128/mbio.01996-22. Page 4, Acknowledgments, lines 3 and 4: “NIH grants or contracts HHSN272201600013C, U01AI151801, and U19AI171413, and. . .” should read “NIH grants HHSN272201600013C and U01AI151801 and by. . .”

