# Supplementary material for: Neutralization Titers in Vaccinated Patients with SARS-CoV-2 Delta Breakthrough Infections
Source: mBio. 2022 Aug 4;13(4):e01996-22. doi: 10.1128/mbio.01996-22 (PMC9426493; doi:10.1128/mbio.01996-22)
Supplement: TABLE S1 [file mbio.01996-22-s0001.docx]

**Supplementary Table 1. Serum information and NT_50_ values.** Serum information including age, gender, ethnicity of patient with vaccine type, and serum collection time (days post positive NAAT and days last vaccination) are indicated. Neutralization titers against WA1 and Delta are shown.

| **Serum ID** | **Age (years)** | **Gender (F/M)** | **Ethnicity** | **Serum collection time (days post positive** ***NAAT)** | **Vaccine type** | ^#^**Days post last vaccination** | ^**NT_50_ (WA1)** | ^**NT_50_ (Delta)** |
| --- | --- | --- | --- | --- | --- | --- | --- | --- |
| 1 | 94 | F | Black | 0 | Pfizer | 137 | ^&^10 | 10 |
| 2 | 74 | F | White | 0 | Pfizer | 176 | 10 | 14 |
| 3 | 74 | F | White | -4 | Pfizer | 193 | 10 | 10 |
| 4 | 91 | F | White | 0 | Pfizer | 203 | 10 | 10 |
| 5 | 83 | F | White | 0 | Pfizer | 182 | 10 | 10 |
| 6 | 66 | M | Black | 0 | Pfizer | 178 | 10 | 10 |
| 7 | 60 | M | Hispanic | 0 | Moderna | 159 | 10 | 10 |
| 8 | 64 | F | Black | 0 | Pfizer | 124 | 10 | 10 |
| 9 | 76 | M | White | 0 | Pfizer | 19 | 10 | 10 |
| 10 | 42 | F | Black | -5 | Pfizer | 216 | 10 | 26 |
| 11 | 53 | F | White | -4 | Pfizer | 149 | 10 | 24 |
| 12 | 50 | M | Black | -1 | Pfizer | 101 | 10 | 12 |
| 13 | 85 | M | White | 0 | Pfizer | 187 | 10 | 10 |
| 14 | 72 | M | Black | 0 | Pfizer | 186 | 10 | 10 |
| 15 | 72 | F | Black | 0 | Pfizer | 166 | 10 | 10 |
| 16 | 88 | M | White | 0 | Moderna | 207 | 10 | 10 |
| 17 | 34 | F | White | -3 | Pfizer | 128 | 10 | 23 |
| 18 | 56 | F | White | 0 | Pfizer | 224 | 10 | 10 |
| 19 | 44 | F | White | 0 | Pfizer | 225 | 10 | 10 |
| 20 | 77 | F | White | 0 | J&J | 179 | 10 | 10 |
| 21 | 97 | F | White | 0 | Pfizer | 205 | 10 | 10 |
| 22 | 54 | F | Black | -1 | Pfizer | 120 | 10 | 10 |
| 23 | 84 | M | White | 0 | Pfizer | 198 | 10 | 23 |
| 24 | 73 | F | White | 0 | Pfizer | 190 | 10 | 10 |
| 25 | 68 | M | White | -3 | Pfizer | 231 | 10 | 10 |
| 26 | 69 | M | White | 0 | Pfizer | 195 | 10 | 10 |
| 27 | 30 | M | White | 0 | Moderna | 156 | 10 | 14 |
| 28 | 52 | F | White | 0 | Pfizer | 98 | 10 | 10 |
| 29 | 69 | F | Black | 0 | J&J | 188 | 10 | 10 |
| 30 | 75 | F | White | 0 | Pfizer | 165 | 10 | 21 |
| 31 | 73 | F | White | 0 | Pfizer | 179 | 10 | 10 |
| 32 | 62 | F | Hispanic | 0 | Pfizer | 225 | 10 | 10 |
| 33 | 68 | F | Black | 0 | J&J | 212 | 10 | 10 |
| 34 | 59 | M | Black | 0 | Pfizer | 149 | 10 | 10 |
| 35 | 80 | M | Hispanic | 0 | J&J | 170 | 10 | 10 |
| 36 | 77 | F | Asian | 0 | Pfizer | 236 | 10 | 10 |
| 37 | 35 | M | White | 0 | Pfizer | 138 | 10 | 10 |
| 38 | 72 | M | White | 0 | Pfizer | 199 | 10 | 10 |
| 39 | 73 | M | White | -5 | Pfizer | 219 | 10 | 10 |
| 40 | 87 | M | White | 0 | Pfizer | 223 | 10 | 10 |
| 41 | 84 | F | Black | 0 | J&J | 211 | 10 | 10 |
| 42 | 85 | F | White | 0 | Pfizer | 241 | 10 | 10 |
| 43 | 78 | F | Hispanic | 0 | Pfizer | 236 | 10 | 10 |
| 44 | 89 | M | White | 0 | Pfizer | 162 | 10 | 15 |
| 45 | 56 | M | Hispanic | 0 | Moderna | 154 | 12 | 25 |
| 46 | 77 | M | White | 0 | Moderna | 238 | 14 | 10 |
| 47 | 77 | M | Black | 0 | J&J | 165 | 14 | 10 |
| 48 | 96 | F | Black | 0 | J&J | 218 | 15 | 22 |
| 49 | 66 | M | White | 0 | Pfizer | 178 | 15 | 20 |
| 50 | 85 | F | Black | 0 | Pfizer | 161 | 16 | 37 |
| 51 | 64 | M | White | 0 | Pfizer | 156 | 19 | 39 |
| 52 | 65 | M | Black | 0 | Pfizer | 113 | 19 | 21 |
| 53 | 30 | M | White | -3 | Pfizer | 223 | 19 | 18 |
| 54 | 62 | M | White | -1 | Pfizer | 175 | 20 | 10 |
| 55 | 60 | F | White | 0 | Pfizer | 41 | 21 | 23 |
| 56 | 16 | F | White | -1 | Pfizer | 129 | 21 | 42 |
| 57 | 20 | F | White | 0 | Pfizer | 119 | 22 | 26 |
| 58 | 41 | F | White | -4 | Pfizer | 190 | 26 | 14 |
| 59 | 34 | F | Hispanic | 0 | Moderna | 163 | 27 | 28 |
| 60 | 61 | M | Native Hawaiian | -3 | Pfizer | 175 | 35 | 52 |
| 61 | 80 | F | White | 0 | Pfizer | 193 | 41 | 53 |
| 62 | 38 | M | Hispanic | 0 | Pfizer | 104 | 41 | 36 |
| 63 | 27 | M | Hispanic | 0 | Pfizer | 145 | 47 | 41 |
| 64 | 38 | F | Hispanic | 0 | Pfizer | 69 | 69 | 69 |

*Nucleic acid amplification test (NAAT)

^#^Days after dose 2 of Pfizer and Moderna vaccine or days after dose 1 of J&J vaccine

^Individual NT_50_ value is the geometric mean of duplicate neutralization test results.

^&^NT_50_ of <20 was treated as 10 for plot purpose and statistical analysis.
